# Phylogenetic diversity and community assembly in a naturally fragmented system

**DOI:** 10.1002/ece3.8404

**Published:** 2021-12-01

**Authors:** Katie Peterson, Megan Ruffley, Christine E. Parent

**Affiliations:** ^1^ Department of Biological Sciences University of Idaho Moscow Idaho USA; ^2^ Department of Plant Biology Carnegie Institution for Science Stanford California USA; ^3^ Institute for Interdisciplinary Data Sciences University of Idaho Moscow Idaho USA

**Keywords:** biodiversity, community ecology, insular communities, phylogenetics, traits, vascular plants

## Abstract

We sought to assess effects of fragmentation and quantify the contribution of ecological processes to community assembly by measuring species richness, phylogenetic, and phenotypic diversity of species found in local and regional plant communities. Specifically, our fragmented system is Craters of the Moon National Monument and Preserve, Idaho, USA. CRMO is characterized by vegetated islands, kipukas, that are isolated in a matrix of lava. We used floristic surveys of vascular plants in 19 kipukas to create a local species list to compare traditional dispersion metrics, mean pairwise distance, and mean nearest taxon distance (MPD and MNTD), to a regional species list with phenotypic and phylogenetic data. We combined phylogenetic and functional trait data in a novel machine‐learning model selection approach, Community Assembly Model Inference (CAMI), to infer probability associated with different models of community assembly given the data. Finally, we used linear regression to explore whether the geography of kipukas explained estimated support for community assembly models. Using traditional metrics of MPD and MNTD neutral processes received the most support when comparing kipuka species to regional species. Individually no kipukas showed significant support for overdispersion. Rather, five kipukas showed significant support for phylogenetic clustering using MPD and two kipukas using MNTD. Using CAMI, we inferred neutral and filtering models structured the kipuka plant community for our trait of interest. Finally, we found as species richness in kipukas increases, model support for competition decreases and lower elevation kipukas show more support for habitat filtering models. While traditional phylogenetic community approaches suggest neutral assembly dynamics, recently developed approaches utilizing machine learning and model choice revealed joint influences of assembly processes to form the kipuka plant communities. Understanding ecological processes at play in naturally fragmented systems will aid in guiding our understanding of how fragmentation impacts future changes in landscapes.

## Graphical abstract

To assess the effects of fragmentation on plant communities at Craters of the Moon National Monument and Preserve in Southern Idaho, USA by measuring species richness, phylogenetic, and phenotypic diversity of species found in local and regional plant communities and quantify the contribution of different ecological processes to community assembly.

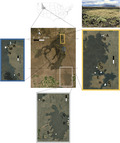

## 1 INTRODUCTION

With the continued anthropogenic alteration of natural landscapes, there is a persistent and pressing need to investigate the consequences of habitat fragmentation and how these consequences affect biodiversity in ecological communities. Specifically, there is a need to understand the effects of fragmentation on phylogenetic and functional trait diversity (Debinski & Holt, 2000; Ewers & Didham, 2006) as they have the power to elucidate past ecological processes that have impacted the community (Cavender‐Bares et al., 2009). Understanding the processes involved in community formation can provide insight into what ecological pressures are influencing community assembly and ultimately the biodiversity we observe (Faith, 1992). By studying recently formed, naturally fragmented landscapes, we can explore the ecological processes that are involved in the early construction of species assemblages, the coexistence of species, and importantly the maintenance of diversity. Thus, if we understand the natural ecological processes at play in response to fragmented landscapes, we can use this information to guide our understanding of how future ecosystems may respond to fragmentation, either natural or human‐caused. Additionally, we can explore the impact of fragmentation on phylogenetic and phenotypic diversity.

Previous work has characterized species richness and phylogenetic diversity in fragmented systems, and sometimes both components are explored (Helm et al., 2006; Santos et al., 2010). In these and other studies, however, the fragmentation process is often implemented experimentally or due to human impacts on a system (Arroyo‐Rodríguez et al., 2012; Laurance et al., 2010). Furthermore, functional trait diversity of fragmented systems is rarely explored alongside phylogenetic information (but see Ribeiro et al., 2017), even though the traits important for existing in a community and local environment can be very telling of the processes that led to the assembly of the current community (de Bello et al., 2009; Kraft et al., 2007; McGill et al., 2006; Weiher & Keddy, 1999). Research has thus far focused on frequency of traits, for example, relative abundance of reproductive strategy and how overall functional diversity is reduced with fragmentation (Girão et al., 2007), rather than the impact of the functional trait variation present. Exploring the effect(s) of fragmentation on phylogenetic and functional trait diversity in a naturally fragmented system will help establish what ecological pressures fragmentation evokes, for example possible increased competition, and how biodiversity is impacted by fragmentation.

The phylogenetic diversity of a community captures information about the amount of evolutionary history shared among the species within a community, which is oftentimes used as a proxy for functional trait differences among species within that particular community (Webb et al., 2002). Phylogenies overall are assumed to reflect morphological, ecological, genetic, and physiological differences that have accumulated between lineages (Gerhold et al., 2015). Phylogenies are thus useful in understanding processes that have influenced, and may continue to influence, multiple aspects of diversity within a community (Brooks & McLennan, 1991; Owen et al., 2019; Tucker et al., 2017; Webb, 2000). For example, community phylogenetic approaches have been used to understand ecological processes important for the assembly of alpine plant communities (Marx et al., 2017), as plants in alpine environments are exposed to harsh conditions requiring a suite of functional traits that may be best represented using a phylogeny.

In the field of community ecology, dispersion metrics calculated from phylogenetic distances between species are often used to infer local ecological processes that have contributed to community structure (Kembel et al., 2010; Kraft et al., 2007; Webb et al., 2002, 2008). The nonneutral processes inferred are generally habitat filtering (Bazzaz, 1991), which is inferred when species within a community are phylogenetically closely related, and competitive exclusion (MacArthur & Levins, 1967), which is inferred from a community of species encompassing high phylogenetic variation (Webb et al., 2008). The justification for these inferences relies on the assumption that most functional traits, especially those important in surviving habitat conditions or local competition for resources, are conserved so that closely related species tend to share similar functional traits. Thus, if many species require similar functional traits to survive in an environment, we then expect these species to be more closely related to one another than by chance, that is, would observe low phylogenetic dispersion. Likewise, if species are competing for a similar niche space, species with traits that are dissimilar are those that exist in the community because they have not outcompeted one another, resulting in species that are not as closely related, and subsequently large phylogenetic dispersion is observed.

In addition to phylogenetic information, morphological, physiological, behavioral, or ecological traits can also be incorporated directly to understand community assembly processes (Cornwell et al., 2006; Kraft et al., 2007, 2015). Traits are often assumed to correlate with phylogenetic information, but this is not always the case (Mazel et al., 2018) and thus sometimes using the traits themselves, rather than the phylogeny as a proxy, can provide a more accurate depiction of community assembly processes (de Bello et al., 2009; Kraft et al., 2007). Specifically, functional trait diversity can, perhaps more directly, provide information about how competition between members in a community might promote or hinder their coexistence (MacArthur & Levins, 1967; McGill et al., 2006; Weiher & Keddy, 1999). Thus, incorporating both phylogenetic and functional trait diversity within a single community can help infer the processes that have led to the assembly of that community, and ultimately what contributes to the maintenance or loss of biodiversity (Cadotte et al., 2013; Webb, 2000).

Utilizing both traits and phylogenies presents challenges, as incorporating both traditional metrics in community ecology is not straightforward. Additionally, the use of phylogenetic dispersion metrics to infer processes of community assembly has presented its own concerns. One of which is the assumption that functional traits important for assembly are conserved across, or correlated with, the phylogeny as this does not always hold (Cavender‐Bares et al., 2009; Mayfield & Levine, 2010) However, limiting analyses to functional traits does not necessarily solve the problem because then the phylogenetic information is not incorporated, meaning information inherent in evolutionary relationships is not accounted for. Additionally, a conclusion based on hypothesis testing and interpretation of processes when a significance threshold is passed is arguably problematic for biological inferences when we know the inference itself exists on a continuum, rather than on a binary threshold (i.e., yes/no). Therefore, we also use an alternative approach, Community Assembly Model Inference (CAMI; Ruffley et al., 2019). This approach attempts to address the aforementioned problems by inferring a model of community assembly using both phylogenetic information and information on a single continuous trait. The advantages of this approach include the avoidance of assumptions as to how traits evolved along a phylogeny and the uncertainty in the community assembly inferences to be quantified, avoiding a significance threshold for inference. In utilizing this new approach, along with traditional dispersion metric approaches, we seek to learn more about the ecological processes at play in naturally fragmented systems by incorporating phylogenetic information and functional traits together.

We ultimately combine the phylogenetic and functional trait data for use in CAMI, a novel machine‐learning model selection approach (Ruffley et al., 2019), to infer the probability associated with different models of community assembly given the data. With CAMI, we also go one step further than testing for nonneutrality by quantifying the strength of proposed nonneutral models associated with inferred processes of community assembly. Finally, with the probabilities associated with the predicted models and their relationship to island meta‐data, such as area and proximity to the outer edge of lava flow, we are able to further quantify the effect of fragmentation on assembly processes. With this information we can ask whether these methods, hypothesis testing with dispersion metrics, and CAMI, are corroborative of each other and whether simultaneously considering phylogenetic and trait information changes the inferences made by dispersion metrics that consider the two methods alone. This work investigates phylogenetic and functional diversity within a naturally fragmented system and ultimately, we assess the effects of fragmentation on kipuka plant communities at Craters of the Moon National Monument and Preserve by (1) measuring species richness, phylogenetic, and phenotypic diversity of species found in the kipuka community and those found in the greater shrub‐steppe region, and (2) quantifying the contribution of different ecological processes to the assembly of communities in the fragmented landscape with both phylogenetic and ecological information. Given the harsh landscape and the isolation of the kipukas, we predict that the assembly of the plant communities in kipukas will be shaped by nonneutral processes, predominantly by environmental conditions and less so by competitive interactions due to the combination of climatic extremes in the availability of water, temperature variation, and high wind experienced in the region.

## 2 METHODS

### 2.1 Study system

Craters of the Moon National Monument and Preserve (CRMO) located in south central Idaho, USA is a naturally fragmented system ideal to explore these questions because the lava‐flow islands of vegetation within the preserve have been formed relatively recently, within the last 15,000 years. The islands are young in age, and there are many of them, thus offering many replicates to detect the impacts of natural fragmentation. Additionally, within CRMO, the plants that exist in the lava‐flow islands experience harsh environmental conditions that have further shaped the assembly of species within the communities. Between 15,000 years ago (kya) and as recently as 2 kya, the eruptive periods at CRMO have resulted in 60 overlapping flows that encompass nearly 1900 km^2^ (Kuntz et al., 1982; National Park Service, 2011). After each eruption, islands of vegetation surrounded by lava flows were formed. These vegetation‐filled lava‐flow islands are known as *kipukas*, a Hawaiian term used for an area of older land that is completely surrounded by an area of younger lava flows (Vandergast & Gillespie, 2004). There are over 500 kipukas at CRMO creating a vegetated archipelago of islands within an “ocean” of basaltic lava. The size of the kipukas ranges from substantially less than one km^2^ up to a privately owned kipuka that is over 341 km^2^ (National Park Service, 2018). The plant communities at CRMO differ depending on successional stage and location, for example whether on lava flows, in cinder areas, or within kipukas.

Plant communities in kipukas are dominated by shrubs like sagebrush (*Artemisia tridentata*) and perennial bunchgrasses such as Idaho Fescue (*Festuca idahoensis*) (Link et al., 2006). Shrubs and perennial bunchgrasses dominate the shrub steppe ecoregion, which covers about 6,450,000 km^2^ of western North America (Daubenmire, 1970; Link et al., 2006; Rickard & Vaughan, 1988). Typical of the semi‐arid shrub steppe ecosystem, the dry climate of CRMO is characterized by a combination of high temperature, low precipitation, and strong winds. Air temperatures approach 30°C in summer months and the surface of the lava can reach 77°C, whereas in the winter the air temperature can get as low as −17°C (NPS Contributors, 1991; Western Regional Climate Center, n.d.). Average annual precipitation ranges throughout the monument from southern portions to northern portions accumulating 38–51 cm, respectively, and most of the precipitation comes in the form of snow (NPS Contributors, 1991). Strong daily afternoon winds are between 24 and 48 km/h (National Park Service, 2016). Individually, these harsh conditions, and the combination of them, limit the possible plant diversity that could persist at CRMO to those species that can deal with these physiological stresses.

For this study, we used data from floristic surveys of vascular plants in 19 kipukas at CRMO. We used the collections, along with a Flora of the shrub‐steppe ecoregion, to describe the phylogenetic diversity in and around the naturally fragmented landscape. We used an existing phylogeny of Spermatophyta (Smith & Brown, 2018) to construct a community‐wide phylogeny of the species in the kipukas and around the region. With these regional and kipuka phylogenies, we used traditional dispersion metrics and hypothesis testing (Webb et al., 2002, 2008) to infer processes of community assembly in the kipukas. As we are interested in the effects of fragmentation on plant communities, we focused on plants collected within kipukas and not on the lava fields.

Plant traits are important for resource acquisition, seed dispersal, reproductive systems, and might be specific adaptations to low water availability. Adaptations include for example, modifications to increase photosynthesis efficiency (e.g., relative abundance of CAM, C3, and C4 species) (Cavagnaro, 1988), a reduction in size of stomata (Sundberg, 1985), and an overall decrease in height to minimize conduit diameter for water transport as a wider diameter makes the species more vulnerable to conduction‐blocking embolisms from drought or cold (Olson et al., 2018). We chose the functional trait of maximum vegetative height to generate phenotypic dispersion metrics as height is a proxy for resource allocation and competitive ability in plants (Cornwell et al., 2014; Weiher & Keddy, 1999; Westoby, 1998). Additionally, it is consistently noted in species descriptions and as such, the amount of missing data would be minimal (Cornwell et al., 2014).

### 2.2 Sampling

**FIGURE 1:**
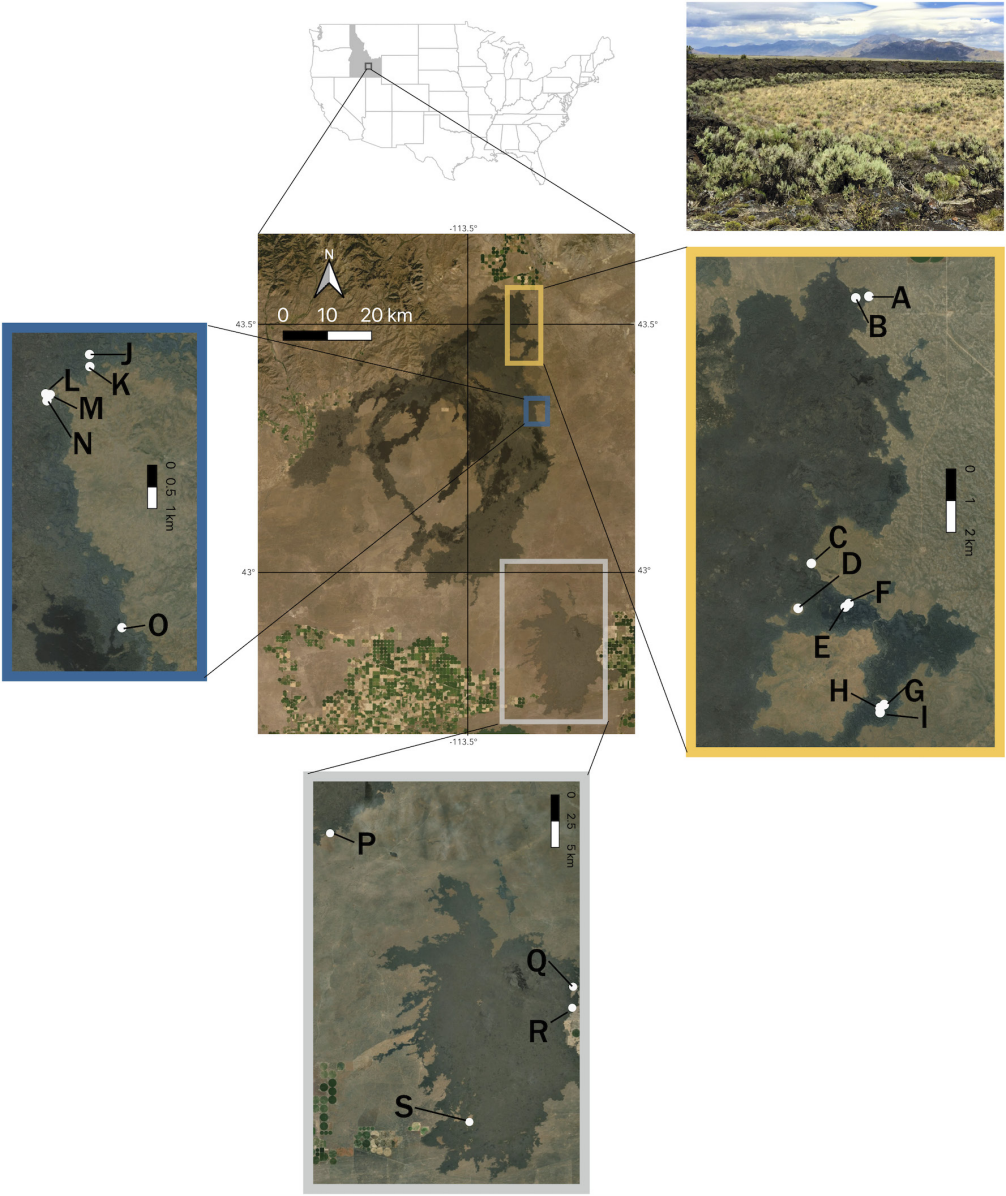
Map of Craters of the Moon National Monument and Preserve, Idaho, USA. Colored outline of map inlays corresponds to organizational scheme of northern, central, and southern regions (yellow, blue, and gray, respectively). The 19 locations of kipukas with vascular plants surveyed are referenced with a letter. Photo at top right is of kipuka “A”

We obtained a permit for specimen collection from the National Park Service. Floristic surveys were conducted in 27 kipukas at CRMO during May–July 2016 and May 2017 (Figure 1). Kipukas were accessed by foot and surveys targeted smaller kipukas that were generally less than 0.02 km^2^ in size where we were confident that the habitat could be thoroughly inventoried by two people in the field by searching within the lava boundary of the kipuka. For each species encountered in a given kipuka, we collected two or three representatives in florescence. Collected plants were pressed, brought back to the University of Idaho for identification, and are stored in the Stillinger Herbarium and publicly available online (www.pnwherbaria.org). The surveys resulted in a total of 66 species, which we use here as the kipuka community species list, and thus used in the kipuka community phylogeny. Nineteen of the 27 kipukas contained nine or more species and were used for subsequent analysis and categorized as northern, central, or southern kipukas (as indicated in Figure 1). We chose that cutoff to keep as many kipukas as possible in our dataset to maximize statistical power while balancing the fact that communities with less than 10 species tend to have error rates in model identification of over 30% (see Ruffley et al., 2019). Identifying and using a comprehensive regional pool is important as this determines the species located within the region that could disperse into the communities of interest. Plant species located up to 17 km away have been demonstrated to have a role in the colonization process after the large‐scale destruction of an ecosystem has occurred (Kirmer et al., 2008). For this study, a regional species pool was compiled by using the kipuka community list and adding the 621 other species listed on existing checklists for vascular plants at CRMO (Popovich, 2006) and the shrub‐steppe ecoregion (Link et al., 2006), resulting in a regional pool, and the regional community phylogeny, consisting of 687 species (Appendix S1). Thus, the kipuka phylogeny is a subset of the regional phylogeny.

### 2.3 Community phylogenetics

We constructed two community phylogenies: one from the species list stemming from all of the kipukas sampled, and one for the regional species pool. This was accomplished by using the *drop*.*tip* and *keep*.*tip* functions in the R package “ape,” “phytools,” and also the grepl function (Paradis et al., 2004; Revell, 2012). The complete regional species pool included all vascular plant species documented within CRMO and the shrub‐steppe ecoregion, as these species are potentially able to colonize the kipukas and thereby play an important role in the colonization process of the kipuka community (Kirmer et al., 2008). We chose to prune from an existing seed plant megaphylogeny (Smith & Brown, 2018) to create a single kipuka phylogeny, as opposed to creating individual community phylogenies for each kipuka, as the approach we chose has been shown to result in a more consistent estimate of evolutionary relationships and distances between taxa (Erickson et al., 2014). We constructed the regional phylogeny in a similar way, by dropping species not included in the regional checklists off of an the seed plant megaphylogeny (Smith & Brown, 2018). This subsampling of the megaphylogeny has the advantage of having no impact on the branch lengths already estimated and recent studies suggest these are reliable trees for community phylogenetic inference (Li et al., 2019). The megaphylogeny we used, which consists of 79,881 vascular plant species with molecular data available from GenBank, is the largest dated phylogeny currently available for seed plants and has broad taxon sampling (Jantzen et al., 2019).

**FIGURE 2:**
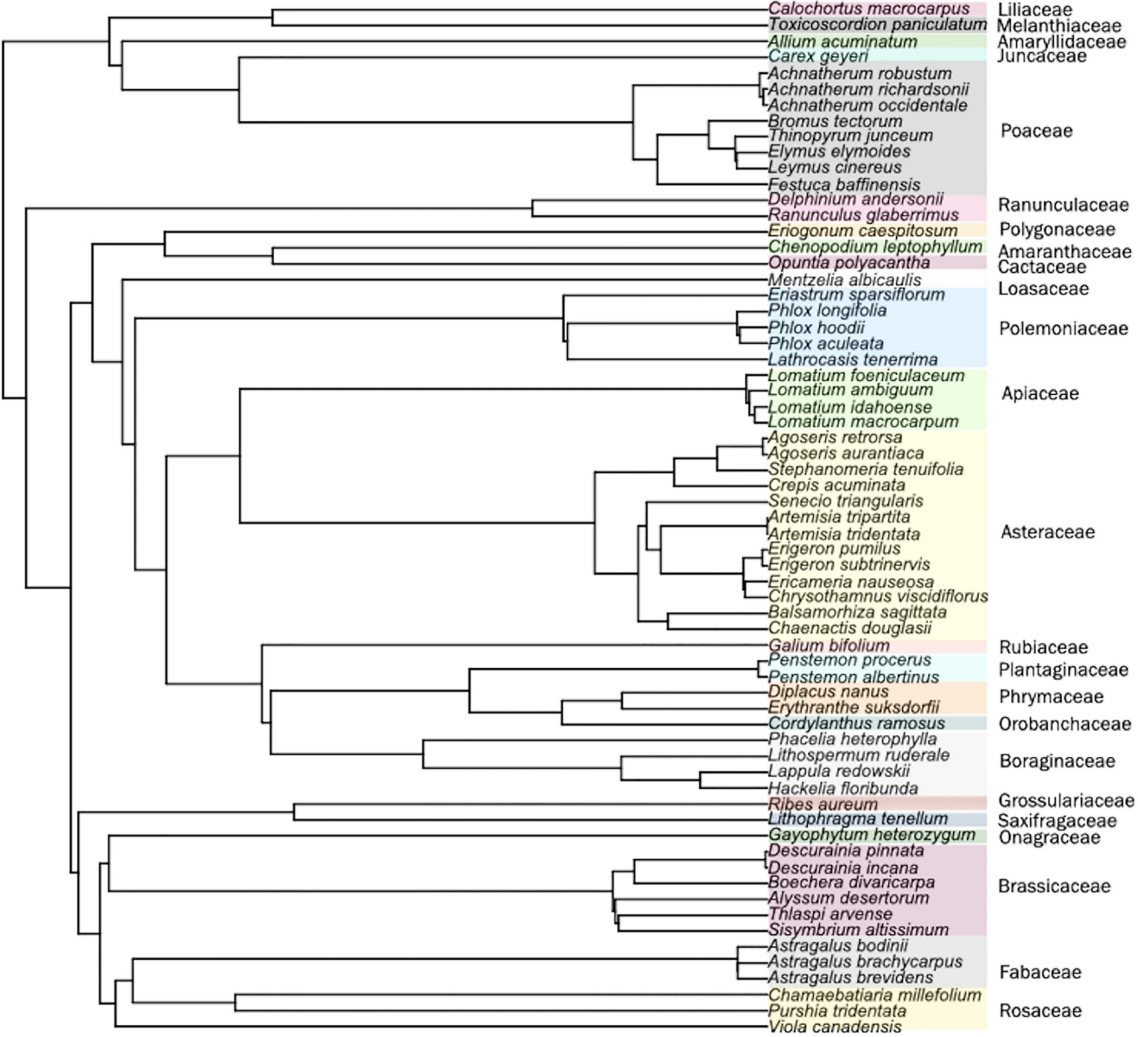
Local community phylogeny of species found in the kipukas sampled at Craters of the Moon National Monument and Preserve, Idaho, USA. Colors shading taxon names correspond to Family listed at right

**FIGURE 3:**
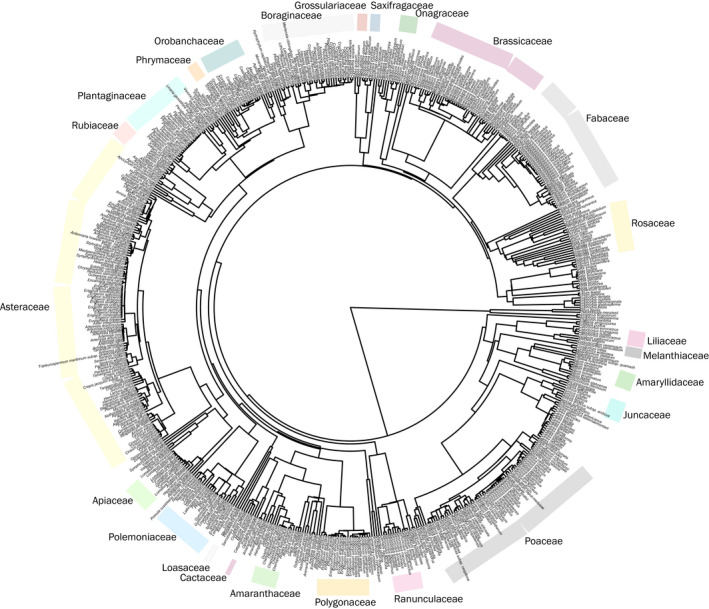
Regional community phylogeny of species found in the shrub‐steppe ecosystem. The bars surrounding the phylogeny loosely indicate Family grouping

If a species was present in the community but absent in the megaphylogeny, a “replacement” species that is a close relative in the same genus with a similar ecological distribution present in the megaphylogeny was retained in the phylogeny (Qian & Jin, 2016). We acknowledge that using replacement species could impact our calculation for community dispersion, though this is unlikely to be significant as a majority of the species relationships are rather distant (Jantzen et al., 2019). Species present in the kipuka and regional communities but for which the genus was not represented in the megaphylogeny and/or no suitable replacement was available (e.g., only one species was present in the megaphylogeny and there were multiple species in the regional species list) were not included in the community phylogenies. The resulting two community phylogenetic trees, after dropping species not present in the checklists and adding replacements, contained 65 and 641 species for the kipukas and regional pool of CRMO, respectively (Figures 2 and 3).

### 2.4 Functional trait

Maximum vegetative height data for all species in the kipuka and regional communities were gathered using a combination of herbarium records, species descriptions, and Floras (Hitchcock & Cronquist, 2018). Maximum vegetative height values were log‐transformed because the data were strongly right skewed. Though it made the data more normal, log transformation was performed primarily for ease of biological interpretation of maximum vegetative height. Notably, a very small number of tree species in the kipukas have very large maximum height values compared to the rest of the species in the kipukas thereby inflating the impact the maximum vegetative height of these species has on the analyses of ecological process. Transforming the data allows us to consider the differences in height at a small scale as equally important as the large differences in height presented by the species of trees within the kipukas.

### 2.5 Community dispersion metrics

We measured the amount of phylogenetic dispersion among species in the kipuka community and tested for significance of the difference between the observed patterns and neutrality by calculating the standardized effect sizes (SES) of two different dispersion metrics (Webb, 2000; Webb et al., 2002) using the R package “picante*”* (Kembel et al., 2010). First, we calculated mean pairwise distance (MPD) between all species in the kipuka community phylogeny. We also calculated the mean nearest taxon distance (MNTD) as the mean distance separating each species in a community from its closest relative, this metric captures how clumped the species in the community are on the phylogenetic tree and the prevalence of short‐branched clusters of species separated by longer branches. We then compared the observed values to the null expectations of these metrics that were produced by generating 1000 replicate metrics. Each of these replicates was made from shuffling the species present in the regional community randomly, resampling the same number of species, and then recalculating the metrics.

If the observed values for MPD or MNTD are significantly under‐dispersed or clustered, the test statistic fell in the lower 2.5% of the values obtained in the null distribution (*p*‐value < .025). A community assembly process of habitat filtering is inferred in this case because the species in the local community are more closely related than is expected by chance (Gotelli & Colwell, 2001; Kembel et al., 2010; Webb, 2000; Webb et al., 2002, 2008). Alternatively, if the observed metrics are significantly over‐dispersed, meaning the test statistic fell within the upper 97.5% of the null distribution (*p*‐value > .0975). In this case, a community assembly process of competitive exclusion is inferred because the species in the local community are more distantly related than you would expect by chance. As these tests are done separately, if neither metric fell in either tail of the null distribution, a neutral process of community assembly was inferred. Though if one metric, either MPD or MNTD was found to be significant and the other not significant, we still considered the significant result.

Mean pairwise distance and MNTD can be calculated using phylogenetic branch lengths, the number of nodal distances, or phenotypic/functional trait differences (Gotelli & Colwell, 2001; Kembel et al., 2010; Webb, 2000; Webb et al., 2002, 2008). Thus, we measured the phenotypic dispersion the same way we calculated the phylogenetic dispersion metrics. We calculated each metric, MPD and MNTD, then performed 1000 random shuffles of the regional and local communities to get the null distribution and to see if the observed metrics fell within either tail. We first ran a comparison between the kipuka and regional communities and then also looked at the kipukas separately by further pruning the phylogeny to represent only species present in a given kipuka. We then repeated this process for each of the remaining kipukas individually.

### 2.6 CAMI

To integrate phenotypic and phylogenetic data while inferring community assembly processes, we used a novel simulation software and inference procedure for community assembly models implemented in the R package “CAMI” (Ruffley et al., 2019). This approach works by first simulating many datasets of phylogenetic and phenotypic data under various community assembly processes such as habitat filtering, competitive exclusion, and neutrality. We then use a set of summary statistics that capture information in the phylogeny and traits to compare the simulations with the observed data. Approximate model selection and parameter estimation methods of random forests (RF; Breiman, 2001) and Approximate Bayesian Computation (ABC; Csilléry et al., 2010) are then used for inference. The simulations used in model selection and the parameter estimation must match the empirical data conditions as much as possible, as described below.

To establish what model under which to simulate data, we first determined the model of trait evolution that best fits the regional phylogeny and regional trait information prior to simulation of phylogenetic and phenotypic data in CAMI. We fit the empirical data to two models of trait evolution, Brownian Motion (BM; Felsenstein, 1985) and Orstein Uhlenbeck (OU; Butler & King, 2004; Hansen, 1997) using the *fitcontinuous()* function in the R package ‘Geiger’ (Pennell et al., 2014). BM models mimic the process of evolutionary drift over macroevolutionary time, *t*, with a single parameter, *σ* ^2^, that controls the rate of phenotypic change through time such that the expected distribution of trait values should be normal with the variance *σ* ^2^ *t*. OU does the same, only it includes a selective regime in which traits are “pulled” toward a phenotypic optimum at a rate of *α*. Using AIC, the best fitting model was found to be OU, which meant both parameters *σ* ^2^ and *α* needed to be estimated. To fit an OU model, we maximized the likelihood of the parameters of the OU model given the kipuka data. However, OU model parameters are notoriously hard to estimate as *σ* ^2^ and *α* are confounded and data can be fit using various combinations of these parameters where the likelihood always gets better with an increasing *α* and smaller *σ* ^2^, though increasing *α* values become more and more unrealistic the larger they get (Uyeda & Harmon, 2014). Therefore, we fit several OU models to the empirical data, varying the bounds of *α* from 0.01 to 1, to determine at what values of *σ* ^2^ and *α* the likelihood stopped getting dramatically better. This was at an estimated *σ* ^2^ of 0.92 with a corresponding estimate of *α* at 0.2; we used these estimates to simulate the trait data in CAMI (Appendix S2).

We simulated 10,000 community assembly datasets for each assembly model, for competitive exclusion, habitat filtering, and neutral, all under an OU model of trait evolution with the above estimated parameters. The other parameters such as the strength of filtering/competition *t* and the phylogenetic parameters, the speciation rate *λ* and the extinction rate *μ*, were drawn from their default uniform prior distributions as implemented in CAMI. The resulting simulated data, along with the empirical data, were summarized into 30 different summary statistics (Appendix S3) to be used for model selection in RF and parameter estimation in ABC.

For community assembly model selection, we constructed a classification forest consisting of 1000 decision trees using the 30,000 simulated datasets and the 30 summary statistics. RF works by using many decision trees to partition out the variation in the summary statistics and uses these differences to distinguish between the three community assembly models. As the decision trees are being constructed, they are also simultaneously being validated by a portion of the data that is withheld from the construction. This enables the calculation of the out‐of‐bag (OOB) error rate, or the proportion of misclassified simulations. This OOB error rate details how accurate the classifier is overall and also for each model, as some models are easier to distinguish than others. The resulting classification forest was then used to determine which model of community assembly structured the kipuka plant communities at CRMO. Here, we inferred the probability of each community assembly model for each of the 19 kipukas surveyed.

We performed parameter estimation using ABC following Ruffley et al. (2019). For ABC, we scaled the summary statistics by their standard deviation and then used the top 10 informative summary statistics from the RF classifier to estimate the posterior probability of *t*, the strength of habitat filtering (Appendix S4). We only considered the simulations under the community assembly model that best fit the data given the RF model selection, (i.e., the habitat filtering simulations). From those, we accepted 100 simulations from the posterior distribution for the parameter *t*. We used these estimates to generate 95% high density confidence intervals (Kruschke, 2011).

### 2.7 Factors influencing community assembly

To understand whether the model probabilities were explained by the fragmented nature of the kipukas, we constructed linear regression models using the *lm()* function in R 3.6.1 and tested whether any significant relationship existed. Specifically, we tested for whether any of the following independent variables; species richness, area of kipuka, distance to the edge of lava flow (isolation), and kipuka elevation, explained the variation in support for community assembly models associated with the 19 kipukas in our study (dependent variable). One may expect the combination of isolation and area, or isolation and elevation to better capture “fragmentation” than just one of the variables alone. Thus, we also tested whether the interaction between any of these variables resulted in a significant relationship with model support. This analysis aimed to understand whether these metrics of fragmentation explained variation in the ecological processes inferred from the phylogenetic and functional trait data.

## 3 RESULTS

### 3.1 Kipuka community diversity and biogeographical attributes

The 66 plant species collected in the 19 kipukas sampled at CRMO represent 24 families and 51 genera. Species richness ranged from nine to 20 species per kipuka. The phylogenies created using an existing seed plant megaphylogeny consisted of 65 and 641 species in the kipuka and in the regional community phylogenies, respectively. Mean maximum vegetative height was 126 cm for the regional community and 77 cm for the kipuka community (Table 1). There was no missing data for the height data for species used in the analysis.

**TABLE 1:** Summary of vegetative height data for the regional community (*n* = 641 species) and the kipuka community (*n* = 65 species) and the biogeographical factors of kipukas that were included in the analyses

	Regional community	Kipuka community
Mean (range) minimum vegetative height (cm)	45 (0.3–4054)	23 (1.3–100)
Mean (range) maximum vegetative height (cm)	126 (1.5–9144)	77 (6–300)
Kipuka mean area (m^2^)	1.37 (700–114,100)	
Kipuka mean isolation (m)	348.5 (17.65–2137)	
Kipuka mean elevation (m)	1574 (1358–1678)	

The mean area of kipukas sampled was 13,670 m^2^, mean kipuka isolation, that is the distance from the edge of a kipuka to the outer lava flow, was 348.5 m, and mean kipuka elevation was 1574 m.

### 3.2 Community dispersion metrics

**FIGURE 4:**
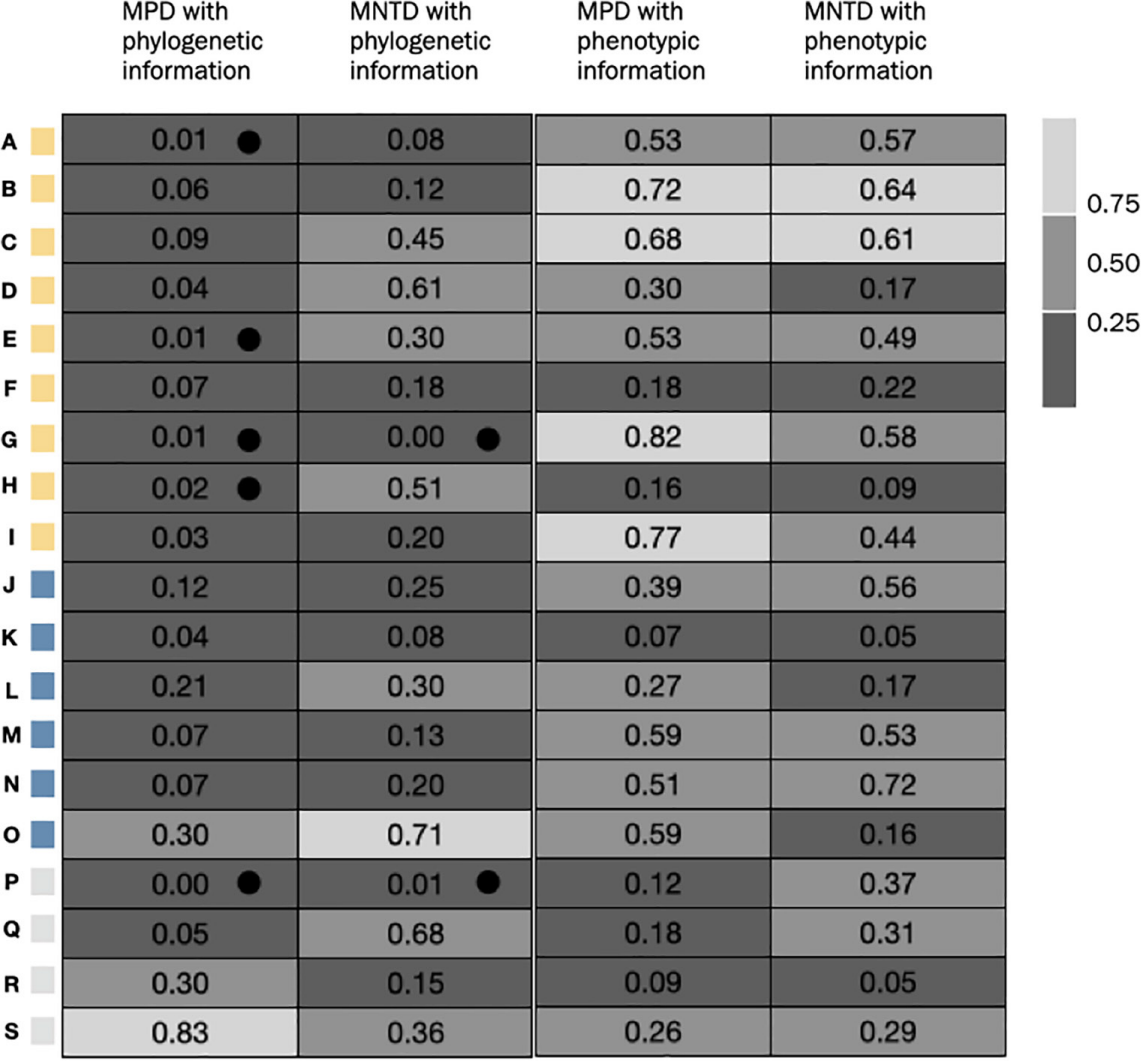
Heatmap of p‐values for the 19 kipukas sampled at Craters of the Moon National Monument and Preserve, Idaho, USA for each phylogenetic and phenotypic diversity metric. The header of each column is the test that the *p*‐value in the cells refers to (mean pairwise distance, MPD and mean nearest taxon distance, MNTD). Colored squares at the left of the heatmap denote the kipuka letter and region (northern, central, and southern) as indicated in Figure 1. Darker gray colors represent lower *p*‐values and lighter gray colors represent higher *p*‐values. The standardized *p*‐value is noted in each cell. Additionally, a black circle within an individual cell represents a *p*‐value of less than .025 indicating significant support for phylogenetic or phenotypic clustering. A *p*‐value of more than .975 would indicate significant support for over‐dispersion

The observed values of MPD and MNTD for the kipuka community as a whole (all 66 species observed in kipukas) suggest that neutral processes are dominant as neither dispersion metric was significantly under‐ or over‐dispersed (Appendix S5). Although not significant, the lower rank of the standardized effect size of the observed MPD (156 out of the 1000 randomizations) shows a tendency to the lower 25% of values (*p*‐value .156) and thus toward an under‐dispersion or clustering signal. The rank of the standardized effect size of the observed MNTD tends toward the middle of randomizations and thus for neutral processes (406 out of 1000). The SES are calculated by standardizing the raw phenotypic and phylogenetic dispersion metrics relative to the total variation observed. The empirically calculated SES is then considered the test statistic when compared to a null distribution of SES and the p‐value is where that test statistic falls within the null distribution. In Figure 4, the p‐values reported in each cell are as follows, for example, kipuka A received a SES rank of 10 out of 1000 randomizations for phylogenetic data using the MPD metric and has a *p*‐value of .01 listed and thus significant support for clustering. In sum, neither process of habitat filtering nor competitive exclusion were inferred with these traditional phylogenetic dispersion metrics.

When considered individually across kipukas, none of the 19 kipukas showed significant support for over‐dispersion with either the MPD or MNTD metric using phylogenetic data (Figure 4). Using the MPD metric based on phylogenetic data, five kipukas showed significant support for phylogenetic clustering and with the MNTD metric based on phylogenetic data, two kipukas showed significant support for phylogenetic clustering. Two kipukas (G and P) showed significant support for clustering with each metric, MPD, and MNTD. Thus, we found more individual kipukas at CRMO to be phylogenetically clustered than over‐dispersed.

Among the remaining 14 kipukas that did not significantly support either clustering or over‐dispersion using the MPD metric and phylogenetic data, eleven trended toward phylogenetic clustering (*p* = .5–.25), and only one (kipuka S) trended toward over‐dispersion (*p* = .75–.95). Using the MNTD metric and phylogenetic data, nine kipukas trended toward clustering. None of the kipukas had significant support for over‐dispersion based on the phylogenetic MNTD metric.

In regard to phenotypic dispersion based on maximum vegetative height and the MPD metric, no kipuka showed significant support for either clustering or over‐dispersion using MPD. Six kipukas had ranks above 50 but less than 250, indicating a trend toward phenotypic clustering. Only two kipukas tended toward over‐dispersion indicating possible competition (kipukas I and G had ranks above 750 but below 950). Nine kipukas trended toward clustering.

### 3.3 Selection of community assembly model

In general, most kipukas had very similar summary statistics, many with an expected amount of deviation given the varying species’ pools across kipukas (Appendix S6 and available at https://github.com/ruffleymr/Peterson_Data/blob/master/KipSummaryStats.csv). Notably, the variance of vegetative height among kipuka species was almost always, except in four kipukas, smaller than that of the regional species pool trait variance. This is somewhat indicative of environmental filtering because the trait variance is decreased in the local community. Specifically, for the sampled kipukas, Blomberg's K, which measures phylogenetic signal, showed there was weak evidence for a phylogenetic signal of the trait maximum vegetative height as the value was generally very low (mean = 0.27, Appendix S5).

**FIGURE 5:**
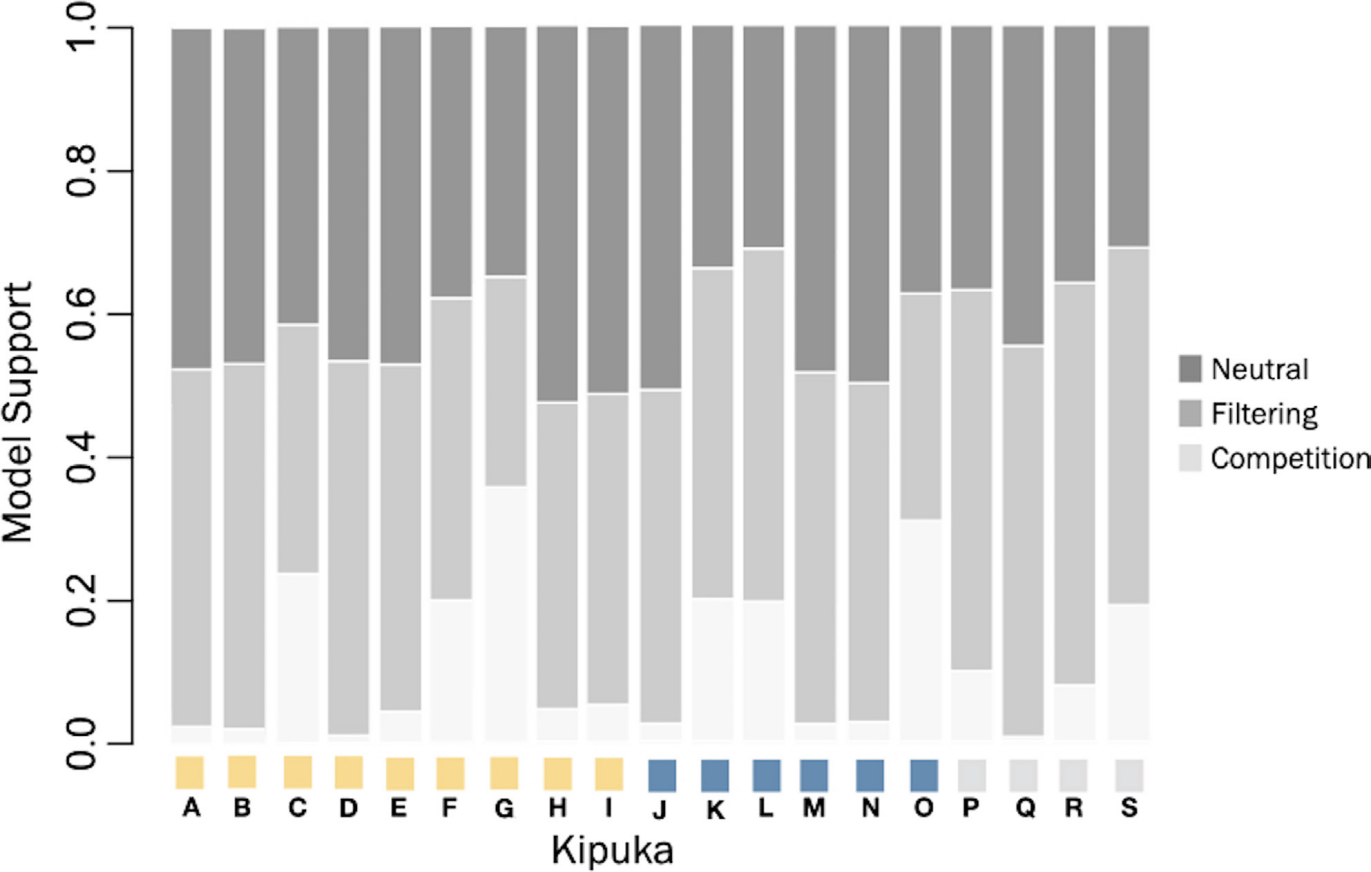
Stacked bar plot of percent model support values for the 19 kipukas. Model support values indicated at left. Colored squares at bottom denote kipuka region (northern, central, and southern as indicated in Figure 1) at Craters of the Moon National Monument and Preserve, Idaho, USA. Shade of bar denotes community assembly model

The classification forest constructed using RF had an overall error rate of 20.23%, meaning that about 20% of the time the classifier is misclassifying simulations into the wrong model of community assembly. More specifically though, with an error rate of 3.1%, the competitive exclusion model was found to have the lowest classification error rate. The other two models, habitat filtering and neutral assembly, had higher error rates of 34% and 25%, respectively, indicating these two models are harder to distinguish from one another but both are easily distinguished from the competition assembly model. Using the classification forest, we were able to infer which model of community assembly structured the kipuka plant community at CRMO by our trait of interest, maximum vegetative height (Figure 5). In general, the competition model had the least support with an average probability of 11% across all kipukas, while the neutral and the filtering models on average had probabilities of 43% and 46%, respectively.

When estimating the *t* parameter, which in this case is the strength of filtering, we simulated under a range of values from one to 60. Counterintuitively, the values closer to 1 indicate strong filtering, while larger values indicate weak filtering. When the values are smaller, the filtering effect is stronger because species are heavily penalized for phenotypes dissimilar to the optimum. The average median estimate of *t* across the kipuka communities was 30.82, ranging from 16.23 to 39.34 (Appendix S7). The 90% high density confidence intervals for the *t* posterior distribution for each of the communities was quite broad, with many of the confidence intervals spanning a majority of the prior distribution.

### 3.4 Factors influencing community assembly

**FIGURE 6:**
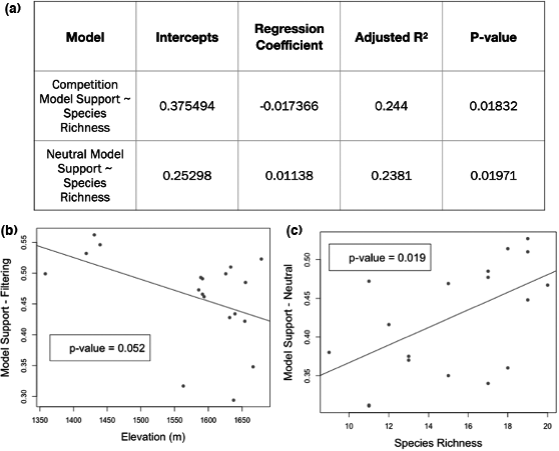
Significant linear regression model results (*α* = 0.05). Top panel (a) includes significant (**) results for the model support (dependent variable) and factor of kipuka (independent variable). Split panels demonstrate (b) nearly significant (*) relationship negative relationship between elevation and model support for filtering and (c) significant (**) positive relationship between species richness and model support for neutrality. The rest of the linear regression model results can be found in Appendix S8

Of all the linear regression models tested to evaluate the effect of kipuka properties on the support for community assembly models, few resulted in significant relationships (*α* = 0.05). The only models with significant prediction ability were species richness predicting model support for competition, as well as species richness predicting model support for the neutral model (Figure 6, Appendix S8). Specifically, as species richness for a kipuka increased, the model support for competition decreases (*p*‐value .018) and the model support for the neutral model of assembly increased (*p*‐value .019). Likewise, elevation was nearly a significant predictor of the model support for habitat filtering (*p*‐value .052), where low elevation kipukas showed higher support for the habitat filtering model (Figure 6). All other models, including those with interaction terms and multiple predictors did not increase the predictability of any of the response variables.

## 4 DISCUSSION

While traditional phylogenetic community approaches based on trait and phylogenetic dispersion suggest neutral assembly dynamics, overall, we do find some support for phylogenetic clustering, and ultimately habitat filtering. Importantly, we find that recently developed approaches utilizing machine learning and model choice in assembly reveal there are joint influences of both neutral dynamics, involving colonization and drift, as well as nonneutral dynamics such as habitat filtering influencing the kipuka plant communities. In combination, these two processes together could be interpreted as mild filtering pressure on the species in the community that are generally under neutral processes. Likewise, we explored the relationships between model support for the various community assembly models, and various factors of fragmentation. Together these analyses allow us to describe the phylogenetic and functional trait diversity across the kipukas and interpret the influence of fragmentation.

### 4.1 Phylogenetic and functional trait diversity

Using traditional dispersion metrics alone, such as MPD and MNTD, and hypothesis testing, our analyses mainly support the role of neutral processes forming the community as very few kipukas resulted in significantly over or under‐dispersed phylogenetic or functional trait metrics. Under a neutral model of assembly, all species present in a regional community pool have an equal probability of colonizing and persisting in that local community (Hubbell, 2001; Rosindell et al., 2012). This neutrality implies that species differences (e.g., in traits) do not impact their presence or absence in the local community. Species neutrality is a main component of the foundational Theory of Island Biogeography (MacArthur & Wilson, 1967), whereas most island systems are a result of who can colonize the open habitat. This may be the case for the kipukas, given their very young age (~15 kya) and the harsh habitat that mimics true island dynamics. Support for neutral processes of community assembly have been found in a variety of isolated and/or fragmented organismal systems including aquatic bacteria communities in tree holes in the same area (Woodcock et al., 2007), farmland birds that exist in a fragmented agricultural landscape (Henckel et al., 2019), and cichlids in Lake Tanganyika (Janzen et al., 2017).

Given that no phylogenetic signal, or Blomberg's K in this case, for our trait of interest was estimated to be of 0.27 across all kipukas, the approaches above were not completely reliable. This is because the use of phylogenetic and functional trait dispersion metrics for community assembly relies on high phylogenetic signal in the trait(s) of interest. Rather an approach that does not assume phylogenetic signal in traits, such as CAMI, is justifiable to use (Cavender‐Bares et al., 2009; Kraft et al., 2007). In CAMI, in all models of community assembly the species in the regional pool have an equal probability of colonizing a community thus, support for neutral and filtering suggests that the trait of maximum vegetative height reflects a barrier for some species inhabiting the kipukas. Perhaps the true functional trait barrier is the height of the plants, or perhaps it is related to the shared resource allocation that the plant trait height is a proxy for. Either way, there is evidence that there is an environmental limitation or barrier to some species existing in the kipukas.

Support for multiple process of community assembly could mean processes of community assembly are operating at different scales. For example, previous work has found multiple mechanisms of community assembly operating in early plant communities (Marteinsdóttir et al., 2018). Assembly from the regional pool to local communities was mostly neutral, and within communities, nonrandom assembly occurred related to various traits important in a plant species ability to disperse, establish, and persist in a local community. Additionally, others have found that different community assembly processes operate at different life stages of plants (Hu et al., 2016). It is important to note that all environments, or each individual kipuka in this case, may not select for the same variant in traits (Lowe & McPeek, 2014). The kipuka community as a whole is then comprised of a set of species that are expressing different traits based on selective pressures at different scales (e.g., spatial, temporal, and phenological) (Hu et al., 2016; Lowe & McPeek, 2014; Marteinsdóttir et al., 2018). Support for both neutral and filtering processes operating in the assembly of the kipuka communities at CRMO may highlight processes impacting at different scales, different life stages, and the differences in selective pressures between kipukas. We may be observing and measuring the initial impacts of fragmentation on the kipuka communities and the long‐term effects of these processes over a macroevolutionary timescale might not yet be realized.

Various traits in plants are important for resource acquisition, seed dispersal, and specific adaptations to the stress of low water availability exist. One of these, a reduction in overall plant height to minimize the diameter of vascular tissue in order to decrease occurrence of embolisms (Olson et al., 2018) would be particularly beneficial in habitats that experience temperature and precipitation extremes, such as at CRMO. We chose the single trait of plant height because of its impact on overall water movement in a plant, as susceptibility to stress due to low water availability and cold would impact a plants ability to persist at CRMO. Water stress in plants has been shown to be an important primary filter in restricting which species present in a regional pool were available to establish via community assembly (Luzuriaga et al., 2012). Future studies including several ecologically relevant traits could reveal a more complete picture of the role of phenotypic variation across species in constraining or promoting the assembly of fragmented communities. Although one quantitative trait can be used at a time in CAMI, multiple analyses could be done to compare across traits.

Qualitative traits, for example seed dispersal mechanisms may vary between plants found within the local kipuka community and those in the regional community (Lowe & McPeek, 2014). Perhaps gravity seed dispersal is more prevalent for the kipuka species than for the regional species, however this was outside of the scope of this study.

In our efforts to measure the strength of filtering through the *t* parameter, we find that we do not have much confidence to estimate this parameter with our current techniques and data. The data are limited by small communities, and we know small communities lead to a lack of power in estimating this parameter (Ruffley et al., 2019). However, we also know that these data support both filtering and neutral models of assembly, which could also be why estimating a parameter only from the filtering model is unsuccessful.

The topography of the kipukas at CRMO could in part explain the support for filtering with our trait of interest, maximum vegetative height, in these fragmented plant communities. In addition to the influence of vegetative height on water conduction in vascular tissue and sensitivity to environmental stressors, susceptibility to wind damage can also determine species presence and persistence in a community. Most of the kipukas are bowl‐shaped with the outer lava flow forming a higher, almost ridge‐like edge and the vegetation within. It might be additionally disadvantageous for plants to be taller than the ridge around the kipuka as high winds could be damaging to the plant. Plants do have the ability to acclimate to wind at multiple scales from cellular to the entire organism, but root or stem failure is still possible (Gardiner et al., 2016). Increased susceptibility and negative impacts of wind damage has been found to be exacerbated when surrounding areas lack vegetation (e.g., denuded) such as those of the lava matrix at CRMO (Laurance & Curran, 2008). Thus, plants with a maximum vegetative height shorter than the lava boundary would be able to withstand the strong winds experienced at CRMO better as they are partially protected within the “bowl” shape.

### 4.2 A fragmented landscape

Within the fragmented landscape of kipukas at CRMO, the trait of maximum vegetative height may be particularly influential in the ability of a species to establish and thrive in the kipukas as height may be especially costly in this environment due to environmental stressors caused by fluctuations of temperature and precipitation that occur. How wind acts as a selective force for plants is of interest in other fragmented landscapes as well, as abiotic factors greatly influence the successful establishment and persistence of a species within a community. The fact that lower elevation kipukas show more support for habitat filtering models compared to the kipuka community as a whole is interesting and could be due to a finer scale filtering pressure along an elevational gradient, in addition to the already mentioned environmental stresses operating on the community as whole.

The impacts of fragmentation can be hard to measure at the phylogenetic scale which broadly characterizes diversity at a macroevolutionary scale. One way to obtain a finer perspective of local diversity within and between kipukas at CRMO for future work could be to incorporate genetic sequencing of individuals from each species collected. Producing species‐specific population genetic data would then allow for quantification of diversity within species and comparisons among species. This proposed population genetic approach would allow us to quantify contemporary migration (i.e., dispersal) occurring within the local community between kipukas. Although outside of the scope of this study, leaf tissue samples were obtained (and stored in silica) from each individual species collected and these could be used in the future in such a proposed population genetic study.

The fragmented landscape due to the lava matrix in which the archipelago of kipukas are situated makes CRMO a particularly useful system in which to ask questions related to functional trait diversity and phylogenetic diversity. Although this system is naturally fragmented, the intervening matrix in many ways is similar to anthropogenic alterations of landscape occurring elsewhere (e.g., asphalt, concrete). By understanding the ecological processes at play in natural fragmented systems and traits that may impact community assembly we can then use this information to guide our conservation and restoration efforts in future fragmented ecosystems.

## 5 CONCLUSION

With the continued alteration to natural landscapes, there is a persistent and pressing need to investigate the consequences of habitat fragmentation and how these consequences may impact phylogenetic and functional trait diversity. The incorporation of both phylogenetic and functional trait diversity within a single community can help infer the processes that have led to the assembly and formation of that community, and ultimately what contributes to the maintenance or loss of biodiversity. Using a new approach that infers a model of community assembly using both phylogenetic and trait information, along with measuring the strength of the inferred ecological process, we find that for the kipuka plant community at CRMO dual processes of neutrality and filtering based on maximum vegetative height have contributed to community formation. Additionally, we find there is evidence that environmental pressures are indeed prohibiting some species from inhabiting some or all of the kipukas, and these pressures may be more severe at lower elevations. When data for more than one trait are available, multiple CAMI analyses could be performed to compare the role of different traits and their impact on community formation. This type of comparative trait‐based analysis could help to predict how community assembly might respond to changes such as fragmentation.

## CONFLICT OF INTEREST

The authors declare no conflict of interest.

## AUTHOR CONTRIBUTIONS

**Katie Peterson:** Conceptualization (equal); Data curation (lead); Formal analysis (equal); Investigation (lead); Methodology (supporting); Writing – original draft (lead); Writing – review & editing (equal). **Megan Ruffley:** Conceptualization (equal); Formal analysis (equal); Methodology (lead); Resources (equal); Software (lead); Writing – review & editing (equal). **Christine E. Parent:** Conceptualization (equal); Formal analysis (equal); Investigation (equal); Resources (lead); Writing – review & editing (equal).

## Supporting information

Appendix S1

Appendix S2

Appendix S3

Appendix S4

Appendix S5

Appendix S6

Appendix S7

Appendix S8

## Data Availability

All input data and scripts for each analysis, along with the output data, can be found in https://github.com/ruffleymr/Peterson_Data and data is located in a permanent Dryad repository https://doi.org/10.5061/dryad.dncjsxm13.

## References

[ece38404-bib-0001] Arroyo‐Rodríguez, V. , Cavender‐Bares, J. , Escobar, F. , Melo, F. P. L. , Tabarelli, M. , & Santos, B. A. (2012). Maintenance of tree phylogenetic diversity in a highly fragmented rain forest. Journal of Ecology, 100(3), 702–711. 10.1111/j.1365-2745.2011.01952.x

[ece38404-bib-0002] Bazzaz, F. A. (1991). Habitat selection in plants. American Naturalist, 137, S116–S130. 10.1086/285142

[ece38404-bib-0003] Breiman, L. (2001). Random Forests. Machine Learning, 45, 5–32.

[ece38404-bib-0004] Brooks, D. R. , & McLennan, D. A. (1991). Phylogeny, ecology, and behavior. A research program in comparative biology. Chicago Press.

[ece38404-bib-0005] Butler, M. A. , & King, A. A. (2004). Phylogenetic comparative analysis: A modeling approach for adaptive evolution. The American Naturalist, 164(6), 683–695. 10.1086/426002 29641928

[ece38404-bib-0006] Cadotte, M. , Albert, C. H. , & Walker, S. C. (2013). The ecology of differences: Assessing community assembly with trait and evolutionary distances. Ecology Letters, 16, 1234–1244. 10.1111/ele.12161 23910526

[ece38404-bib-0007] Cavagnaro, J. B. (1988). Distribution of C3 and C4 grasses at different altitudes in a temperate arid region of Argentina. Oecologia, 76, 273–277. 10.1007/BF00379962 28312206

[ece38404-bib-0008] Cavender‐Bares, J. , Kozak, K. H. , Fine, P. V. A. , & Kembel, S. W. (2009). The merging of community ecology and phylogenetic biology. Ecology Letters, 12(7), 693–715. 10.1111/j.1461-0248.2009.01314.x 19473217

[ece38404-bib-0009] Cornwell, W. K. , Schwilk, D. W. , & Ackerly, D. D. (2006). A trait‐based test for habitat filtering: Convex hull volume. Ecology, 87, 1465–1471. 16869422 10.1890/0012-9658(2006)87[1465:attfhf]2.0.co;2

[ece38404-bib-0010] Cornwell, W. K. , Westoby, M. , Falster, D. S. , FitzJohn, R. G. , O'Meara, B. C. , Pennell, M. W. , McGlinn, D. J. , Eastman, J. M. , Moles, A. T. , Reich, P. B. , Tank, D. C. , Wright, I. J. , Aarssen, L. , Beaulieu, J. M. , Kooyman, R. M. , Leishman, M. R. , Miller, E. T. , Niinemets, Ü. , Oleksyn, J. , … Zanne, A. E. (2014). Functional distinctiveness of major plant lineages. Journal of Ecology, 102, 345–356. 10.1111/1365-2745.12208

[ece38404-bib-0011] Csilléry, K. , Blum, M. G. B. , Gaggiotti, O. E. , & François, O. (2010). Approximate Bayesian Computation (ABC) in practice. Trends in Ecology & Evolution, 25, 410–418. 10.1016/j.tree.2010.04.001 20488578

[ece38404-bib-0012] Daubenmire, R. (1970). Steppe vegetation of Washington. Technical Bulletin. Washington Agricultural Experiment Station, 62, 131.

[ece38404-bib-0013] de Bello, F. , Thuiller, W. , Lepš, J. , Choler, P. , Clément, J.‐C. , Macek, P. , Sebastià, M.‐T. , & Lavorel, S. (2009). Partitioning of functional diversity reveals the scale and extent of trait convergence and divergence. Journal of Vegetation Science, 20, 475–486. 10.1111/j.1654-1103.2009.01042.x

[ece38404-bib-0014] Debinski, D. M. , & Holt, R. D. (2000). A Survey and Overview of Habitat Fragmentation Experiments Sondeo y Revisión de Experimentos de Fragmentación de Hábitat. Conservation Biology, 14(2), 342–355. 10.1046/j.1523-1739.2000.98081.x

[ece38404-bib-0015] Erickson, D. L. , Jones, F. A. , Swenson, N. G. , Pei, N. , Bourg, N. A. , Chen, W. , Davies, S. J. , Ge, X.‐J. , Hao, Z. , Howe, R. W. , Huang, C.‐L. , Larson, A. J. , Lum, S. K. Y. , Lutz, J. A. , Ma, K. , Meegaskumbura, M. , Mi, X. , Parker, J. D. , Fang‐Sun, I. , … Kress, W. J. (2014). Comparative evolutionary diversity and phylogenetic structure across multiple forest dynamics plots: A mega‐phylogeny approach. Frontiers in Genetics, 5, 1–14. 10.3389/fgene.2014.00358 25414723 PMC4220724

[ece38404-bib-0016] Ewers, R. M. , & Didham, R. K. (2006). Confounding factors in the detection of species responses to habitat fragmentation. Biological Reviews of the Cambridge Philosophical Society, 81(1), 117–142. 10.1017/S1464793105006949 16318651

[ece38404-bib-0017] Faith, D. P. (1992). Conservation evaluation and phylogenetic diversity. Biological Conservation, 61(1), 1–10. 10.1016/0006-3207(92)91201-3

[ece38404-bib-0018] Felsenstein, J. (1985). Phylogenies and the comparative method. American Naturalist, 125, 1–15. 10.1086/284325 31094602

[ece38404-bib-0076] Gardiner, B. , Berry, P. , & Moulia, B. (2016). Wind impacts on plant growth, mechanics and damage. Plant Science, 245, 94–118. 26940495 10.1016/j.plantsci.2016.01.006

[ece38404-bib-0019] Gerhold, P. , Cahill, J. F. , Winter, M. , Bartish, I. V. , & Prinzing, A. (2015). Phylogenetic patterns are not proxies of community assembly mechanisms (they are far better). Functional Ecology, 29, 600–614. 10.1111/1365-2435.12425

[ece38404-bib-0020] Girão, L. C. , Lopes, A. V. , Tabarelli, M. , & Bruna, E. M. (2007). Changes in tree reproductive traits reduce functional diversity in a fragmented Atlantic forest landscape. PLoS One, 2(9), e908. 10.1371/journal.pone.0000908 17878943 PMC1975471

[ece38404-bib-0021] Gotelli, N. J. , & Colwell, R. K. (2001). Quantifying biodiversity: Procedures and pitfalls in the measurement and comparison of species richness. Ecology Letters, 4, 379–391. 10.1046/j.1461-0248.2001.00230.x

[ece38404-bib-0022] Hansen, F. (1997). Stabilizing selection and the comparative analysis of adaptation. Evolution, 51, 1341–1351. 10.1111/j.1558-5646.1997.tb01457.x 28568616

[ece38404-bib-0023] Helm, A. , Hanski, I. , & Pärtel, M. (2006). Slow response of plant species richness to habitat loss and fragmentation. Ecology Letters, 9(1), 72–77. 10.1111/j.1461-0248.2005.00841.x 16958870

[ece38404-bib-0074] Henckel, L. , Meynard, C. N. , Devictor, V. , Mouquet, N. , & Bretagnolle, V. (2019). On the relative importance of space and environment in farmland bird community assembly. PLoS One, 14(3), 1–19. 10.1371/journal.pone.0213360 PMC6411160 30856193

[ece38404-bib-0024] Hitchcock, C. L. , & Cronquist, A. C. (2018). Flora of the Pacific Northwest: An illustrated manual (D. E. Giblin, B. S. Legler, P. F. Zika, & R. G. Olmstead, Eds.) (2nd ed.). University of Washington Press.

[ece38404-bib-0025] Hu, G. , Feeley, K. J. , & Yu, M. (2016). Habitat fragmentation drives plant community assembly processes across life stages. PLoS One, 11(7), e0159572. 10.1371/journal.pone.0159572 27427960 PMC4948860

[ece38404-bib-0071] Hubbell, S. P. (2001). The unified neutral theory of biodiversity and biogeography. Princeton University Press. 10.1016/j.tree.2011.03.024 21561679

[ece38404-bib-0026] Jantzen, J. R. , Whitten, W. M. , Neubig, K. M. , Majure, L. C. , Soltis, D. E. , & Soltis, P. S. (2019). Effects of taxon sampling and tree reconstruction methods on phylodiversity metrics. Ecology and Evolution, 9, 9479–9499. 10.1002/ece3.5425 31534670 PMC6745870

[ece38404-bib-0075] Janzen, T. , Alzate, A. , Muschick, M. , Maan, M. E. , van der Plas, F. , & Etienne, R. S. (2017). Community assembly in Lake Tanganyika cichlid fish: Quantifying the contributions of both niche‐based and neutral processes. Ecology and Evolution, 7(4), 1057–1067. 10.1002/ece3.2689 28303177 PMC5306054

[ece38404-bib-0027] Kembel, S. W. , Cowan, P. D. , Helmus, M. R. , Cornwell, W. K. , Morlon, H. , Ackerly, D. D. , Blomberg, S. P. , & Webb, C. O. (2010). Picante: R tools for integrating phylogenies and ecology. Bioinformatics, 26, 1463–1464. 10.1093/bioinformatics/btq166 20395285

[ece38404-bib-0028] Kirmer, A. , Tischew, S. , Ozinga, W. A. , von Lampe, M. , Baasch, A. , & van Groenendael, J. M. (2008). Importance of regional species pools and functional traits in colonization processes: Predicting re‐colonization after large‐scale destruction of ecosystems. Journal of Applied Ecology, 45, 1321–1329. 10.1111/j.1365-2664.2007.0

[ece38404-bib-0029] Kraft, N. J. B. , Cornwell, W. K. , Webb, C. O. , & Ackerly, D. D. (2007). Trait evolution, community assembly, and the phylogenetic structure of ecological communities. The American Naturalist, 170, 271–283. 10.1086/519400 17874377

[ece38404-bib-0030] Kraft, N. J. B. , Godoy, O. , & Levine, J. M. (2015). Plant functional traits and the multidimensional nature of species coexistence. Proceedings of the National Academy of Sciences of the United States of America, 112, 797–802. 10.1073/pnas.1413650112 25561561 PMC4311865

[ece38404-bib-0031] Kruschke, J. K. (2011). Bayesian assessment of null values via parameter estimation and model comparison. Perspectives on Psychological Science, 6(3), 299–312. 10.1177/1745691611406925 26168520

[ece38404-bib-0032] Kuntz, M. A. , Champion, D. E. , Spiker, E. C. , Lefebvre, R. H. , & McBroome, L. A. (1982). The Great Rift and the evolution of the craters of the Moon Lava Field, Idaho. Cenezoic Geology of Idaho: Idaho Bureau of Mines and Geology Bulletin, 26, 423–437. Retrieved from http://geology.isu.edu/Digital_Geology_Idaho/papers/B‐26ch7‐2.pdf

[ece38404-bib-0033] Laurance, S. G. W. , Laurance, W. F. , Andrade, A. , Fearnside, P. M. , Harms, K. E. , Vicentini, A. , & Luiza, R. C. C. (2010). Influence of soils and topography on Amazonian tree diversity: A landscape‐scale study. Journal of Vegetation Science, 21, 96–106. 10.1111/j.1654-1103.2009.01122.x

[ece38404-bib-0034] Laurance, W. F. , & Curran, T. J. (2008). Impacts of wind disturbance on fragmented tropical forests: A review and synthesis. Austral Ecology, 33, 399–408. 10.1111/j.1442-9993.2008.01895.x

[ece38404-bib-0035] Li, D. , Trotta, L. , Marx, H. E. , Allen, J. M. , Sun, M. , Soltis, D. E. , Soltis, P. S. , Guralnick, R. P. , & Baiser, B. (2019). For common community phylogenetic analyses, go ahead and use synthesis phylogenies. Ecology, 100(9), 1–15. 10.1002/ecy.2788 PMC7079099 31225900

[ece38404-bib-0036] Link, S. O. , Mast, W. H. , & Hill, R. W. (2006). Shrub steppe restoration. In D. Apostol & M. Sinclair (Eds.), Restoring the Pacific Northwest: The art and science of ecological restoration in Cascadia (pp. 216–240). Island Press.

[ece38404-bib-0037] Lowe, W. H. , & McPeek, M. A. (2014). Is dispersal neutral? Trends in Ecology and Evolution, 29(8), 444–450. 10.1016/j.tree.2014.05.009 24962790

[ece38404-bib-0038] Luzuriaga, A. L. , Sánchez, A. M. , Maestre, F. T. , & Escudero, A. (2012). Assemblage of a semi‐arid annual plant community: Abiotic and biotic filters act hierarchically. PLoS One, 7(7), 1–9. 10.1371/journal.pone.0041270 PMC3407229 22848455

[ece38404-bib-0039] MacArthur, R. , & Levins, R. (1967). The limiting similarity, convergence, and divergence of coexisting species. American Naturalist, 101, 377–385. 10.1086/282505

[ece38404-bib-0040] Marteinsdóttir, B. , Svavarsdóttir, K. , & Thórhallsdóttir, T. E. (2018). Multiple mechanisms of early plant community assembly with stochasticity driving the process. Ecology, 99(1), 91–102. 10.1002/ecy.2079 29121406

[ece38404-bib-0041] Marx, H. E. , Dentant, C. , Renaud, J. , Delunel, R. , Tank, D. C. , & Lavergne, S. (2017). Riders in the sky (islands): Using a mega‐phylogenetic approach to understand plant species distribution and coexistence at the altitudinal limits of angiosperm plant life. Journal of Biogeography, 44, 2618–2630. 10.1111/jbi.13073 29249850 PMC5730081

[ece38404-bib-0070] Mayfield, M. M. , & Levine, J. M. (2010). Opposing effects of competitive exclusion on the phylogenetic structure of communities. Ecology Letters, 13, 1085–1093. 20576030 10.1111/j.1461-0248.2010.01509.x

[ece38404-bib-0042] Mazel, F. , Pennell, M. W. , Cadotte, M. W. , Diaz, S. , Dalla Riva, G. V. , Grenyer, R. , Leprieur, F. , Mooers, A. O. , Mouillot, D. , Tucker, C. M. , & Pearse, W. D. (2018). Prioritizing phylogenetic diversity captures functional diversity unreliably. Nature Communications, 9(1), 1–9. 10.1038/s41467-018-05126-3 PMC6056549 30038259

[ece38404-bib-0043] McGill, B. J. , Enquist, B. J. , Weiher, E. , & Westoby, M. (2006). Rebuilding community ecology from functional traits. Trends in Ecology & Evolution, 21, 178–185. 10.1016/j.tree.2006.02.002 16701083

[ece38404-bib-0044] National Park Service . (2011). Listing of Acreage (Summary). Retrieved from https://irma.nps.gov/STATS/FileDownload/107

[ece38404-bib-0045] National Park Service . (2016). Weather. Retrieved from https://www.nps.gov/crmo/learn/nature/weather.htm

[ece38404-bib-0046] National Park Service . (2018). Kipukas. Retrieved from https://www.nps.gov/crmo/learn/nature/kipukas.htm

[ece38404-bib-0047] NPS Contributors . (1991). Craters of the Moon: National Park Handbook (139). National Park Service.

[ece38404-bib-0048] Olson, M. E. , Soriano, D. , Rosell, J. A. , Anfodillo, T. , Donoghue, M. J. , Edwards, E. J. , León‐Gómez, C. , Dawson, T. , Camarero Martínez, J. J. , Castorena, M. , Echeverría, A. , Espinosa, C. I. , Fajardo, A. , Gazol, A. , Isnard, S. , Lima, R. S. , Marcati, C. R. , & Méndez‐Alonzo, R. (2018). Plant height and hydraulic vulnerability to drought and cold. Proceedings of the National Academy of Sciences of the United States of America, 115(29), 7551–7556. 10.1073/pnas.1721728115 29967148 PMC6055177

[ece38404-bib-0049] Owen, N. R. , Gumbs, R. , Gray, C. L. , & Faith, D. P. (2019). Global conservation of phylogenetic diversity captures more than just functional diversity. Nature Communications, 10(1), 8–10. 10.1038/s41467-019-08600-8 PMC6382770 30787282

[ece38404-bib-0050] Paradis, E. , Claude, J. , & Strimmer, K. (2004). APE: Analyses of phylogenetics and evolution in R language. Bioinformatics, 20(2), 289–290. 10.1093/bioinformatics/btg412 14734327

[ece38404-bib-0051] Pennell, M. W. , Eastman, J. M. , Slater, G. J. , Brown, J. W. , Uyeda, J. C. , FitzJohn, R. G. , Alfaro, M. E. , & Harmon, L. J. (2014). geiger v2.0: an expanded suite of methods for fitting macroevolutionary models to phylogenetic trees”. Bioinformatics, 30, 2216–2218. 10.1093/bioinformatics/btu181 24728855

[ece38404-bib-0052] Popovich, S. J. (2006). Craters of the Moon vascular plant checklist. Retrieved from https://www.nps.gov/crmo/learn/nature/upload/CRMO_Final_2006_Plant_Checklist_10‐15‐06.pdf

[ece38404-bib-0053] Qian, H. , & Jin, Y. (2016). An updated megaphylogeny of plants, a tool for generating plant phylogenies and an analysis of phylogenetic community structure. Journal of Plant Ecology, 9(2), 233–239. 10.1093/jpe/rtv047

[ece38404-bib-0054] Revell, L. J. (2012). phytools: An R package for phylogenetic comparative biology (and other things). Methods in Ecology and Evolution, 3(2), 217–223. 10.1111/j.2041-210X.2011.00169.x

[ece38404-bib-0055] Ribeiro, J. , Colli, G. R. , Batista, R. , & Soares, A. (2017). Landscape and local correlates with anuran taxonomic, functional and phylogenetic diversity in rice crops. Landscape Ecology, 32(8), 1599–1612. 10.1007/s10980-017-0525-8

[ece38404-bib-0056] Rickard, W. H. , & Vaughan, B. E. (1988). Plant community characteristics and responses. In W. H. Rickard , L. E. Rogers , B. E. Vaughan , & S. F. Liebetrau (Eds.), Shrub‐steppe: Balance and change in a semi‐arid terrestrial ecosystem (pp. 109–181). Elsevier Science.

[ece38404-bib-0072] Rosindell, J. , Hubbell, S. P. , He, F. , Harmon, L. J. , & Etienne, R. S. (2012). The case for ecological neutral theory. Trends in Ecology and Evolution, 27(4), 203–208. 10.1016/j.tree.2012.01.004 22341498

[ece38404-bib-0057] Ruffley, M. , Peterson, K. , Week, B. , Harmon, L. J. , & Tank, D. C. (2019). Identifying models of trait‐mediated community assembly using random forests and approximate Bayesian computation. Ecology and Evolution, 9, 13218–13230. 10.1002/ece3.5773 31871640 PMC6912896

[ece38404-bib-0058] Santos, A. , Arroyo‐Rodriguez, V. , Moreno, C. E. , & Tabarelli, M. (2010). Edge‐related loss of tree phylogenetic diversity in the severely fragmented Brazilian Atlantic Forest. PLoS One, 5(9), e12625. 10.1371/journal.pone.0012625 20838613 PMC2935881

[ece38404-bib-0059] Smith, S. A. , & Brown, J. W. (2018). Constructing a broadly inclusive seed plant phylogeny. American Journal of Botany, 105(3), 302–314. 10.1002/ajb2.1019 29746720

[ece38404-bib-0060] Sundberg, M. D. (1985). Trends in distribution and size of stomata in desert plants. Desert Plants, 7(3), 154–157.

[ece38404-bib-0061] Tucker, C. M. , Cadotte, M. W. , Carvalho, S. B. , Davies, T. J. , Ferrier, S. , Fritz, S. A. , Grenyer, R. , Helmus, M. R. , Jin, L. S. , Mooers, A. O. , Pavoine, S. , Purschke, O. , Redding, D. W. , Rosauer, D. F. , Winter, M. , & Mazel, F. (2017). A guide to phylogenetic metrics for conservation, community ecology and macroecology. Biological Reviews, 92(2), 698–715. 10.1111/brv.12252 26785932 PMC5096690

[ece38404-bib-0062] Uyeda, J. , & Harmon, L. (2014). A novel Bayesian method for inferring and interpreting the dynamics of adaptive landscapes from phylogenetic comparative data. Systematic Biology, 63(6), 902–918. 10.1093/sysbio/syu057 25077513

[ece38404-bib-0063] Vandergast, A. G. , & Gillespie, R. G. (2004). Effects of natural forest fragmentation on a Hawaiian spider community. Environmental Entomology, 33(5), 1296–1305. 10.1603/0046-225X-33.5.1296

[ece38404-bib-0064] Webb, C. O. (2000). Exploring the phylogenetic structure of ecological communities: An example for rain forest trees. The American Naturalist, 156, 145–155. 10.1086/303378 10856198

[ece38404-bib-0065] Webb, C. O. , Ackerly, D. D. , & Kembel, S. W. (2008). Phylocom: Software for the analysis of phylogenetic community structure and trait evolution. Bioinformatics, 24, 2098–2100. 10.1093/bioinformatics/btn358 18678590

[ece38404-bib-0066] Webb, C. O. , Ackerly, D. D. , McPeek, M. A. , & Donoghue, M. J. (2002). Phylogenies and community ecology. Annual Review of Ecology and Systematics, 33(1), 475–505. 10.1146/annurev.ecolsys.33.010802.150448

[ece38404-bib-0067] Weiher, E. , & Keddy, P. (1999). Assembly rules as general constraints on community composition. In E. Weiher , & P. Keddy (Eds.), Ecological assembly rules: Perspectives, advances, retreats (pp. 251–271). Cambridge University Press.

[ece38404-bib-0068] Western Regional Climate Center . (n.d.). Craters of the Moon, Idaho. Retrieved from https://wrcc.dri.edu/cgi‐bin/cliMAIN.pl?id2260

[ece38404-bib-0069] Westoby, M. (1998). A leaf‐height‐seed (LHS) plant ecology strategy scheme. Plant and Soil, 199, 213–227. 10.1023/A:1004327224729

[ece38404-bib-0073] Woodcock, S. , Van Der Gast, C. J. , Bell, T. , Lunn, M. , Curtis, T. P. , Head, I. M. , & Sloan, W. T. (2007). Neutral assembly of bacterial communities. FEMS Microbiology Ecology, 62(2), 171–180. 10.1111/j.1574-6941.2007.00379.x 17937674

