# Supplementary material for: Phylogenetic diversity and community assembly in a naturally fragmented system
Source: Ecol Evol. 2021 Dec 1;11(24):18066–80. doi: 10.1002/ece3.8404 (PMC8717291; doi:10.1002/ece3.8404)
Supplement: Supplementary file 3 — Appendix S3 [file ECE3-11-18066-s007.docx]

**Supplemental Table 3.** Table of summary statistics from Community Assembly Model Inference (CAMI, from Ruffley et al. 2019).
