# Supplementary material for: Phylogenetic diversity and community assembly in a naturally fragmented system
Source: Ecol Evol. 2021 Dec 1;11(24):18066–80. doi: 10.1002/ece3.8404 (PMC8717291; doi:10.1002/ece3.8404)
Supplement: Supplementary file 8 — Appendix S8 [file ECE3-11-18066-s002.docx]

**Supplemental Table 8**. Results of linear regression models. Testing if the biogeographical attributes of the kipukas explained the variation in support for the community assembly models (** significant result, * nearly significant result, α = 0.05).

| **Model** | **Intercept** | **Regression coefficient** | **Adjusted R^2^** | **P-value** |
| --- | --- | --- | --- | --- |
| Competition ~ species richness | 0.375494 | -0.017366 | 0.244 | 0.01832** |
| Neutral ~ species richness | 0.25298 | 0.01138 | 0.2381 | 0.01971** |
| Filtering ~ species richness | 0.371524 | 0.005990 | 0.01968 | 0.2594 |
| Competition ~ kipuka elevation | 1.027e-01 | 6.071e-06 | -0.05879 | 0.983 |
| Neutral ~ kipuka elevation | -0.1241236 | 0.0003482 | 0.1595 | 0.05083* |
| Filtering ~ kipuka elevation | 1.0214410 | -0.0003543 | 0.1567 | 0.05252* |
| Competition ~ kipuka area | 1.0135 | -2.3329 | 0.06012 | 0.1678 |
| Neutral ~ kipuka area | -1.996 | 7.747 | -0.007796 | 0.3665 |
| Filtering ~ kipuka area | -2.612 | 8.462 | 0.005019 | 0.3109 |
| Competition ~ distance to outside lava | 316.06 | -689.98 | 0.0366 | 0.2178 |
| Neutral ~ distance to outside lava | -256.5 | 1153.5 | 0.04411 | 0.2003 |
| Filtering ~ distance to outside lava | -1.322 | 527.734 | -0.03548 | 0.5273 |
